# Highly-sensitive detection of *Salmonella typhi* in clinical blood samples by magnetic nanoparticle-based enrichment and *in-situ* measurement of isothermal amplification of nucleic acids

**DOI:** 10.1371/journal.pone.0194817

**Published:** 2018-03-28

**Authors:** Avinash Kaur, Arti Kapil, Ravikrishnan Elangovan, Sandeep Jha, Dinesh Kalyanasundaram

**Affiliations:** 1 Centre for Biomedical Engineering, Indian Institute of Technology Delhi, New Delhi, India; 2 Department of Microbiology, All India Institute of Medical Sciences, New Delhi, India; 3 Department of Biochemical Engineering and Biotechnology, Indian Institute of Technology Delhi, New Delhi, India; 4 Department of Biomedical Engineering, All India Institute of Medical Sciences, New Delhi, India; University of Helsinki, FINLAND

## Abstract

Enteric fever continues to be a major cause of mortality and morbidity globally, particularly in poor resource settings. Lack of rapid diagnostic assays is a major driving factor for the empirical treatment of enteric fever. In this work, a rapid and sensitive method ‘*Miod*^’^ ‘has been developed. *Miod* includes a magnetic nanoparticle-based enrichment of target bacterial cells, followed by cell lysis and loop-mediated isothermal amplification (LAMP) of nucleic acids for signal augmentation along with concurrent measurement of signal via an *in*–*situ* optical detection system. To identify positive/negative enteric fever infections in clinical blood samples, the samples were processed using *Miod* at time = 0 hours and time = 4 hours post-incubation in blood culture media. Primers specific for the STY2879 gene were used to amplify the nucleic acids isolated from *S*. *typhi* cells. A limit of detection of 5 CFU/mL was achieved. No cross-reactivity of the primers were observed against 10^6^ CFU/mL of common pathogenic bacterial species found in blood such as *E*. *coli*, *P*. *aeruginosa*, *S*. *aureus*, *A*. *baumanni*, *E*. *faecalis*, *S*. *Paratyphi A and K*. *pneumonia*. *Miod* was tested on 28 human clinical blood samples. The detection of both pre-and post-four-hours incubation confirmed the presence of viable *S*. *typhi* cells and allowed clinical correlation of infection. The positive and negative samples were successfully detected in less than 6 hours with 100% sensitivity and specificity.

## Introduction

*Salmonella typhi* causes the enteric fever that is a major public health problem, both in developing as well as developed economies [1,2]. About 21 million cases and 222,000 deaths per year are caused worldwide by *Salmonella typhi* [3]. Antibiotics such as ampicillin, chloramphenicol, cotrimoxazole, fluoroquinolones and 3^rd^ generation cephalosporin are the antibiotics of choice for the treatment of enteric fever. However, typhoidal *Salmonella* species have increasingly become resistant to conventional antibiotics such as ampicillin, chloramphenicol, cotrimoxazole, and fluoroquinolones in developing countries [4]. The death rate in enteric fever is expected to increase by 30% without appropriate diagnosis and effective antibiotic therapy [5].

Blood culture remains the gold standard test for diagnosis of enteric fever till today. The organism once isolated from culture assay is further identified by biochemical tests [6,7]. Serological methods such as Widal test are regularly employed in many healthcare settings [8]. However, these serological methods have low sensitivity and specificity and are inconclusive [9]. So far, confirmation of enteric fever depends on isolation of *Salmonella typhi* from the clinical specimens such as urine, bone marrow, rose spot extracts, duodenal aspirates and stool [10]. Blood culture based diagnosis of enteric fever demands (a) multiple time-consuming protocols (b) skilled labour and (c) numerous instruments and related infrastructure. Further, only 45 to 70% of true positive cases are identified by this method [11]. These challenges in *S*. *typhi* diagnosis are amplified multifold especially in resource-poor settings. Also, the lack of specific, rapid and affordable diagnostic assays lead to inappropriate use of antibiotics in all fevers cases, majority of which are viral or malaria. In addition to the above facts, approximately 20 to 30 mL of blood is required for detection of blood-related infections (both aerobic and anaerobic bacterial cultures). Requirement of such volumes of blood pose a challenge for geriatric and neonatal patients [12]. A diagnostic method requiring minimal blood volumes with rapid and accurate detection is therefore required.

Some of the recently published methods for the detection of *S*. *typhi* in blood, water samples, and food are, lateral flow immunoassay (LFIA) using antibody-coated gold nanoparticles [13], uniplex and, multiplex PCR [14], reverse transcriptase multiplex PCR (RT-MPCR) [15] and gel electrophoresis [16]. The limit of detection (LOD) of these above methods ranges between 500 to 10^4^ CFU/mL. Besides, these methods are time-consuming, require numerous equipments including expensive thermocyclers and trained labour. Henceforth, there is a necessity for new methods that offer rapid, specific, and sensitive detection.

Isothermal amplification of nucleic acids is a promising option for quick and effective amplification eliminating the need for multiple cycles of rapid heating and cooling as demanded in thermocycling PCR [17]. This feature greatly reduces the complexity of the device and therefore the cost. Hence, isothermal based techniques have the potential for easy implementation in developing economies. Various isothermal amplification methods have been introduced over the last decade, such as, nucleic acid sequence-based amplification (NASBA) [18], loop-mediated amplification (LAMP) [19,20], strand displacement amplification [21], helicase-dependent amplification (HDA) [17], rolling circle amplification (RCA) [22], recombinase polymerase amplification (RPA) [23], and multiple displacement amplification (MDA) [24]. Of the above techniques, LAMP assay utilizes four to six primers along with strand displacing DNA polymerase and amplifies target sequences in a rapid manner besides achieving high specificity. LAMP assay can be utilized for many applications including detection of pathogens in food products, environmental samples, genetic testing, and point-of-care testing [25]. Cost-effective devices have been intended to enhance the portability of the LAMP assay for field applications.

Ravan and Yazdanparast reported LAMP–ELISA assay for the detection of enteric fever in spiked samples. The spiked samples were incubated up to 24 hours. The hybridization of probe and amplification were performed simultaneously. The spiked samples were detected in 2 hours 40 minutes by measuring absorbance at 450 nm using microplate ELISA reader. The LOD of this assay was reported as 10 CFU/mL [26]. Bozorgmehr et al. used LAMP based non-crosslinking gold nanoprobes for the detection of *S*. *typhi* DNA. The team used surface plasmon resonance (SPR) for end point-detection [27]. However, in the two methods described above, no clinical blood samples were reported. Abdullah et al. developed an in-house LAMP assay for the detection of *S*. *typhi* using three sets of primers designed for PapD gene. LAMP reaction was performed using heating block (at 63°C for 60 minutes) followed by detection using colorimetry. The LAMP method was compared against the gold standard of culture method and polymerase chain reaction (PCR). The team reported a LOD of 10^4^ CFU/mL or 20 CFU/reaction while that of PCR was 200 CFU/reaction [28].

The sensitivity of LAMP assay can be improved considerably by an enrichment protocol. Immunomagnetic concentration and separation of target bacteria offers an advantage over labour- intensive conventional pathogen enrichment methods (such as biological or serological confirmation, plating and enrichment methods) [29]. Though LAMP is sensitive at lower copies of nucleic acids, however, it is prone to inhibition and contaminants that are often found in clinical samples. Immunomagnetic isolation helps to remove contaminants that might interfere with the subsequent LAMP amplification detection assay [30,31]. In other words, the enrichment protocol enhances the LAMP process by (a) eliminating of the contaminants (thereby reducing the incidence of false positive and false negative test results [29]) and by (b) concentrating the target cells in a smaller volume. The utilization of 100 nm magnetic nanoparticles (MNPs) enhances the binding kinetics in a diffusion-limited process [32]. Likewise, a higher surface to volume proportion enhances the signal by bringing down the steric obstruction during ligand-receptor binding due to bigger molecule surface curvature [33]. *Miod* was developed in the present study that employs a magnetic nanoparticle-based enrichment protocol followed by isothermal amplification of *S*. *typhi* nucleic acids and encompasses an *in-situ* optical detection system with high specificity. In the preceding work, the protocol for magnetic nanoparticle-based enrichment of *S*. *typhi* was optimized. The capture efficiency of *S*. *typhi* cells using antibody coated MNPs was found to be above 65% [29]. In the current work, isothermal amplification and detection were augmented to create a stand-alone system. For isothermal amplification or signal augmentation, the STY2879 gene was amplified using 4 primary primers (pair of inner primers (FIP and BIP) and pair of outer primers (F3 and B3)) along with 2 additional loop primers (LF and LB). Gene STY2879 encodes for reverse transcriptase protein in all *S*. *typhi* isolates. The specificity and cross-reactivity were tested against other bacterial isolates such as *Escherichia coli*, *Staphylococcus aureus*, *Pseudomonas aeruginosa*, *Acinetobacter baumanni*, *Enterococcus faecalis*, *Salmonella Paratyphi A* and *Klebsiella pneumonia*. *Miod* was evaluated against 28 *human clinical blood samples* of suspected enteric fever patients.

## Materials and methods

### Institute ethical approval

This study was approved by ethics committee of All India Institute of Medical Sciences, New Delhi (document number IEC-307 dated 07^th^ June 2016). Blood samples were obtained from all participants with a written consent form, at the department of microbiology of All India Institute of Medical Sciences, New Delhi. The blood samples were spiked in blood culture media immediately.

### Materials used

Dextran-coated magnetic nanoparticles (MNPs) of 100 nm with free carboxyl groups at the surface (FluidMAG-CMX, Chemicell, Germany) conjugated with the polyclonal antibody against *Salmonella spp*. (Rabbit antisera, Difco™, BD) were used for the enrichment of *S*. *typhi* cells. Isothermal LAMP master mix (Optigene, UK), and primers (Integrated DNA Technologies, US) were used for the nucleic acid amplification. Reagents such as phosphate buffer saline (PBS), tryptone soy broth (TSB), bovine serum albumin (BSA), tween 20, 100X Tris-EDTA (TE) buffer, N-(3-dimethylaminopropyl)-N′-ethylcarbodiimide hydrochloride (EDC), N-hydroxysuccinimide (NHS) were obtained from Sigma-Aldrich, USA. Neodymium magnets with approximately 300 mT surface field were procured from a local hardware store. *S*. *typhi* Ty2 (ATCC 19430) strain, available with the department of microbiology at All India Institute of Medical Sciences was used for standardization.

### Bacterial culture for standardization

*S*. *typhi* was grown on TSB for 12 hours at 37°C. The cells were collected in the mid-log phase of the culture (O.D. ~ 0.6, approximately corresponding to 5×10^8^ CFU/mL). The correlation between optical density and CFU/mL was obtained via serial dilution and plating method. The cells were spiked in sterile blood culture media and diluted serially to obtain 500, 400, 300, 200, 100, 50, 10 and 5 CFU/mL. All serial dilutions were carried out using sterile blood culture media. The mixtures were vortexed for 30 seconds for homogenous mixing. The sterile blood culture media without spiking of *S*. *typhi* was used as negative control. One milliliter solution of each concentration was used for obtaining the first standardization curve. The spiked blood cultures were incubated for 4 hours at 37°C for growth of the *S*. *typhi* cells. Post incubation, one milliliter of a solution of each concentration was used for obtaining the second standardization curve. To obtain the concentration of *S*. *typhi* for pre- and post-four-hour incubation, 20 μL of each dilution was plated on MacConkey^®^ agar plates followed by colony counts after 24 hours.

### Conjugation of magnetic nanoparticles with *S*. *typhi* specific antibodies

Antibody conjugated magnetic nanoparticles (MNP-Ab) were prepared as described elsewhere [29]. In brief, MNPs were washed twice with 10 mM PBS, followed by incubation with 20 mg/mL of EDC and NHS for 15 minutes at room temperature (25°C). MNPs were washed again twice with PBS and then incubated with 50 μg of antibodies (Rabbit antisera, Difco™, BD) for 12 hours at room temperature. To remove non-specifically bound antibodies, the MNPs were washed with 1% BSA and 0.1% Tween 20 for 1 hour at room temperature and stored at 4°C.

### MNP based enrichment, cell lysis and DNA isolation

**Fig 1:**
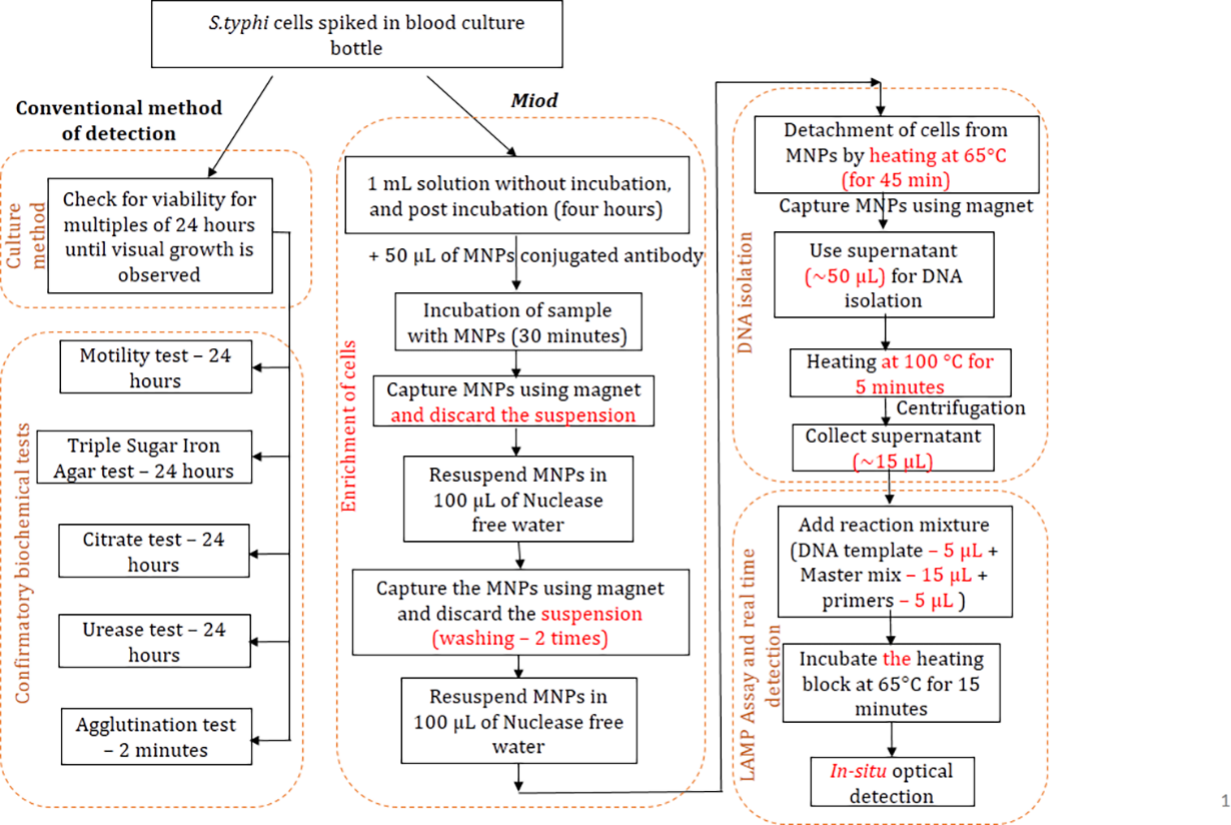
Flowchart showing detail protocol for conventional method and DNA based detection using LAMP.

*S*. *typhi* cells were enriched using MNPs-Ab in the ratio of 20:1 (i.e. 1 mL of solution to 50 μL of MNPs-Ab). After 30 minutes of incubation at room temperature, target bacteria from the blood culture media bound to the MNPs via the antibodies, were isolated using magnets held on the outside of the vial. The remaining contents in the samples were eliminated by washing twice with nuclease-free water (NFW). The enrichment process helps to eliminate the contaminants that hinder the LAMP amplification process. The concentrated cells were finally resuspended in 50 μL of NFW. The suspension was heated at 65°C for 45 minutes, to allow detachment of MNPs from target *S*. *typhi* cells. The supernatant containing the cells were heat-treated at 100°C for 5 minutes for cell lysis. Post cell lysis, the suspension was centrifuged at 12,000 RPM for 5 minutes. Five microlitres of the supernatant were collected and used for the LAMP assay. The protocol is shown in Fig 1.

### Nucleic acid amplification

LAMP based amplification mandates four primers; forward outer primer (F3), backward outer primer (B3), forward inner primer (FIP), backward inner primer (BIP) along with two additional primers; loop forward (LF) and loop backward (LB) primers. Loop primers enhance the rate of amplification. Detailed LAMP method of amplification is described elsewhere [34]. LAMP assay reaction was performed in 25 μL (total volume) reaction mixture containing 15 μL of LAMP master mix, 5 μL of primer mix (0.2 μM of F3 and B3 primers, 0.8 μM of FIP and BIP, 0.4 μM of LF and LB primers) and 5 μL of target or template DNA. The LAMP master mix contains Geobacillus species DNA polymerase (Optigene, UK), optimized reaction buffer containing Mg_2_Cl_2_, deoxynucleotide triphosphates and ds-DNA binding dye (Optigene, UK). The FIP, BIP, F3 and B3 primers for the STY2879 gene were obtained from Fan et al.[35]. The loop primers LF and LB were designed using a commercial software LAMP designer software version 1.10 (Optigene, UK).

### Standardization of concentration versus intensity

LAMP reactions were carried out for different dilutions of *S*. *typhi* cells spiked in sterile blood culture media from 500 CFU/mL down to 5 CFU/mL, pre—and post-incubation (4 hours). DNA from each dilution was pre-concentrated using MNPs-Ab and lysed using heat treatment method. Five microlitres of the supernatant were collected and used for the LAMP assay. LAMP reaction was carried out at 65°C for 15 minutes. The fluorescence intensity during LAMP reaction mix increased over time, as detected by the fiber optic reflectance probe and a fiber optic spectrometer (HR 2000+ES, Ocean optics, USA). The intensity corresponding to each concentration was measured at different time intervals of 5, 10, 15, 20, 25 and 30 minutes. The intensity grew clearly distinguishable between various concentrations at 15 minutes. An increase in the intensity between pre—and post incubated samples confirms cell viability. The intensity at 520 nm of wavelength was obtained for the pre-and post—incubated samples for various concentration at 15 minutes to plot the calibration curve (or standard curve). All experiments were carried out in triplicates.

### Cross-reactivity and specificity with common pathogens/nuclei acids

Common pathogenic bacterial species including *E*. *coli*, *S*. *aureus*, *P*. *aeruginosa*, *A*. *baumanni*, *E*. *faecalis*, *S*. *paratyphi A and K*. *pneumonia* are also typically found in blood samples of enteric fever patients. Hence, experiments on the cross-reactivity of the primers along with master mix and 10^6^ CFU/mL concentration of the above bacterial species were carried out. DNA was isolated using the protocol as described earlier. All experiments were carried out in triplicates.

### LAMP assay from clinical samples

Human clinical blood samples from 28 suspected enteric fever patients were collected at the medical institute. Upon collection of blood from the patient, the blood is immediately mixed with culture media in the blood culture bottles. The blood collection was carried out using standard protocol as described elsewhere [36,37]. Briefly, the site of venipuncture was disinfected using 70% alcohol, 10% povidone iodine and again 70% alcohol. Five milliliters of blood were drawn from the patient. It was then spiked in blood culture bottles containing 45 mL of brain heart infusion (BHI) media (BD Difco, USA). The bottle was incubated at 37°C for culture. DNA was isolated after enrichment of cells using MNPs-Ab from 1 mL of media before and incubation. The cells were then lysed using heat treatment method as described in the MNP based Enrichment, cell lysis and DNA isolation section. Five microlitres of the supernatant were collected and used for the LAMP assay. LAMP assay was run for both pre- and post-four hour incubated samples.

### Comparison with conventional method

The results of *Miod* were validated by conventional methods. First, the blood culture bottle samples were incubated at 37°C for the culture of the bacterial organisms for 24 hours and 48 hours until observation of turbidity. The samples were further sub-cultured on a MacConkey agar plates (BioMesrieux, France) at 37°C for 24 hours. The formation of pale transparent colonies indicates the possible presence of *S*. *typhi* as per the standard protocol discussed elsewhere [36,37]. Confirmatory biochemical test such as motility test, triple sugar iron (TSI) test, urease test, citrate test and slide agglutination test were performed post sub-culturing on agar plate. Motility test was used to determine the motility of *S*. *typhi* cells. Triple sugar iron agar test was used to determine whether S. typhi cells utilize glucose, lactose or sucrose and produce hydrogen sulfide (H_2_S) gas. Citrate utilization test was used to determine the ability of *S*. *typhi* cells to utilize sodium citrate as a carbon source and inorganic NH_4_H_2_PO_4_ as a source of nitrogen. Urease test was used to determine whether *S*. *typhi* cells produce urease enzyme, an indication of viable cells. A slide agglutination test using Antisera (Statens serum institute, Copenhagen) was used to detect *S*. *typhi* O9, poly O and H antigens in blood. A brief description of each of the biochemical tests are included in the supplementary section S1.2 and shown in S1 Fig. The conventional method in total consumes a minimum of 72 hours as compared to *Miod’s* protocol of less than 6 hours, as given in S1 Table.

#### Statistical analysis

Based on the outcomes, the clinical sensitivity, specificity, positive predictive value (PPV), and negative predictive value (NPV) were calculated to estimate the effectiveness and efficiency of *Miod* [38].

## Results and discussions

### Design of device

**Fig 2:**
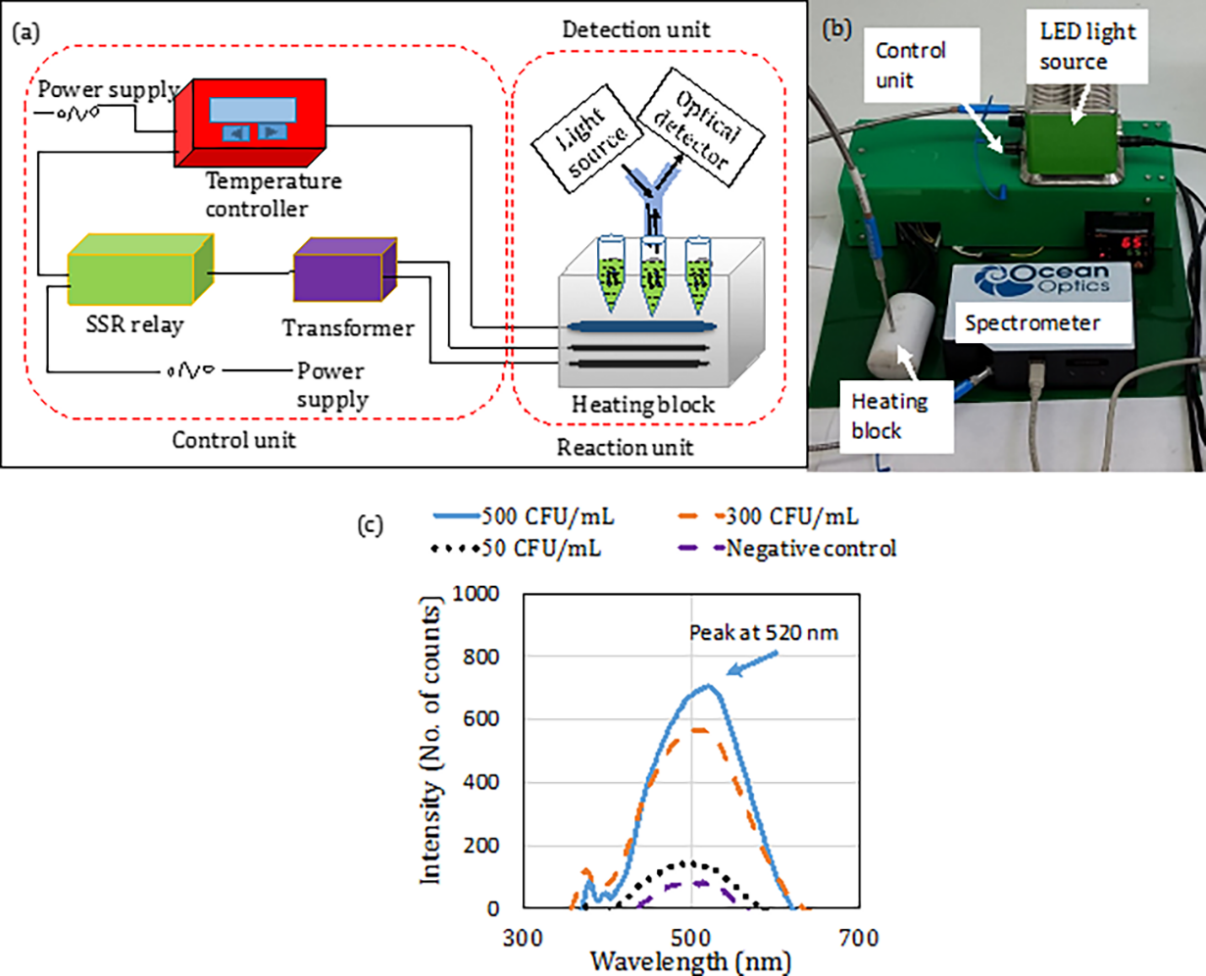
(a) Schematic and (b) actual view: isothermal amplification and detection assembly (c) signal at detector at various bacterial concentrations.

An aluminum heating block of dimensions (length 5 cm, breadth 3 cm, height 2.5 cm) was machined with three wells to accommodate conical PCR tubes of 200 μL. The depth and diameter of the well was 10 mm and 5.5 mm. Three horizontal bores of diameters 6 mm and length 50 mm were drilled to seat two cartridge type heaters (Pratik heat products Pvt. Ltd., Mumbai, India) and one thermocouple (Pixsys electronics, Italy). The thermocouple was placed underneath the wells of PCR tubes to monitor the temperature of the wells during the reaction. The cartridge heaters were placed parallel to both sides of the wells. Electrical power to the heaters and thermocouple were supplied by two 1-Ampere transformers connected in parallel. Thermocouple was linked to corresponding integral derivative (PID) controller (Selec, Mumbai) through solid state relay (SSR) (FUTEK, USA) to control the temperature of the heating block. LAMP assay was carried out at 65° C for 15 minutes. The reaction was then terminated by denaturing the polymerase in the master mix (Optigene, UK) by heating to 80° C for 2 minutes. The setup along with the instrumentation is shown in Fig 2. The current dimension of the device is 30 cm × 30 cm × 8 cm. Y-type optical probes were placed on top of the vials in the heating block. The optical fiber transmits white light from the source (Ocean optics, USA) into the vials. The fluorescence light intensity (at a shorter band of wavelength) was observed at the flip side of the optic fiber utilizing a spectrometer (HR 2000+ES, Ocean optics, USA) and spectra suite software.

### Standardization of intensity versus concentration

**Fig 3:**
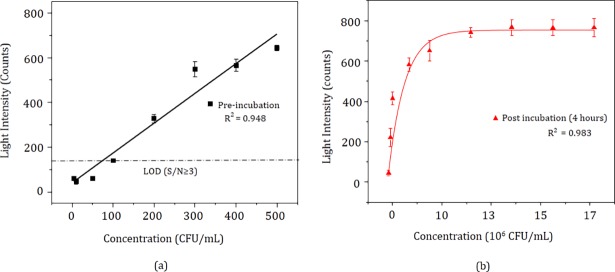
**Calibration curve**: Intensity at 15 minutes (a) LAMP assay versus initial concentration from 5 to 500 CFU/mL along with (b) post-four-hour incubation. For post-incubated samples, the approximate CFU/mL count was obtained by a count of cultured bacteria on agar plates as shown by red lines. The linear regime is shown by the dotted lines.

The intensity of fluorescence was measured *in-situ* during LAMP reaction. At 15 minutes and at 520 nm of wavelength, the intensity for pre-incubation and post-four-hour incubation are shown in Fig 3(A) and 3(B) respectively. The R^2^ value for pre-incubation was 0.948 and for post-incubation (4 hours) was 0.983. The response of the detector for the negative control was found to be 45 counts and 57 counts for pre-incubation and post-four-hour incubation respectively. The theoretical limit of detection (LOD) calculated at 3 times the sensor reading of negative control are 135 counts (for pre-incubation), and 171 counts (for post-four incubation) for reliable detection of the signal. Hence, based on *S*/*N* ratio ≥3, the LOD of pre-incubated samples was 200 CFU/mL (corresponding to 327 counts) while in the case of post-four-hour incubation, the LOD was 5 CFU/mL (corresponding to 221 counts). The response of the detector was linear at lower concentrations (5 to 500 CFU/mL) and non-linear at higher concentrations as shown in Fig 3(A) and 3(B) respectively. Linear curve fitting was used to fit the response for pre-incubated samples. The equation of best-line-fit was *x* = [(*y*-38.20)/1.331] where, *x* represents the concentration in CFU/mL and *y* represents the intensity in number of counts. The Boltzmann equation for post-four-hour incubated samples was [*x* = 39.70 ln ((*y*+5.28E6)/ (754.84-*y*))–354.20] respectively. This represents nonlinear curve fitting. An unknown concentration of the cells in a given sample can be determined by using the equation of the standard curve. The results of the bacterial count were obtained by plating (S2 Table and S2 Fig).

Our protocol involves experimenting both pre-incubated and post-incubated samples to ascertain the viability of the cells in addition to high sensitivity. The initial concentration of 5 CFU/mL, yielded an intensity of 221 counts post-four-hour incubation. Hence, we conclude that our theoretical LOD is 5 CFU/mL. The saturation for the post-incubation curve is due to the limited number of fluorescent molecules. Since the experiment is designed to detect one lakh *S*. *typhi* cells as a limit of detection shown in Fig 3. In the linear regime, the slope of the calibration curve for pre-incubation sample was 1.331 counts per CFU/mL which, represents the sensitivity of the sensor.

### Cross-reactivity tests for primers

**Fig 4:**
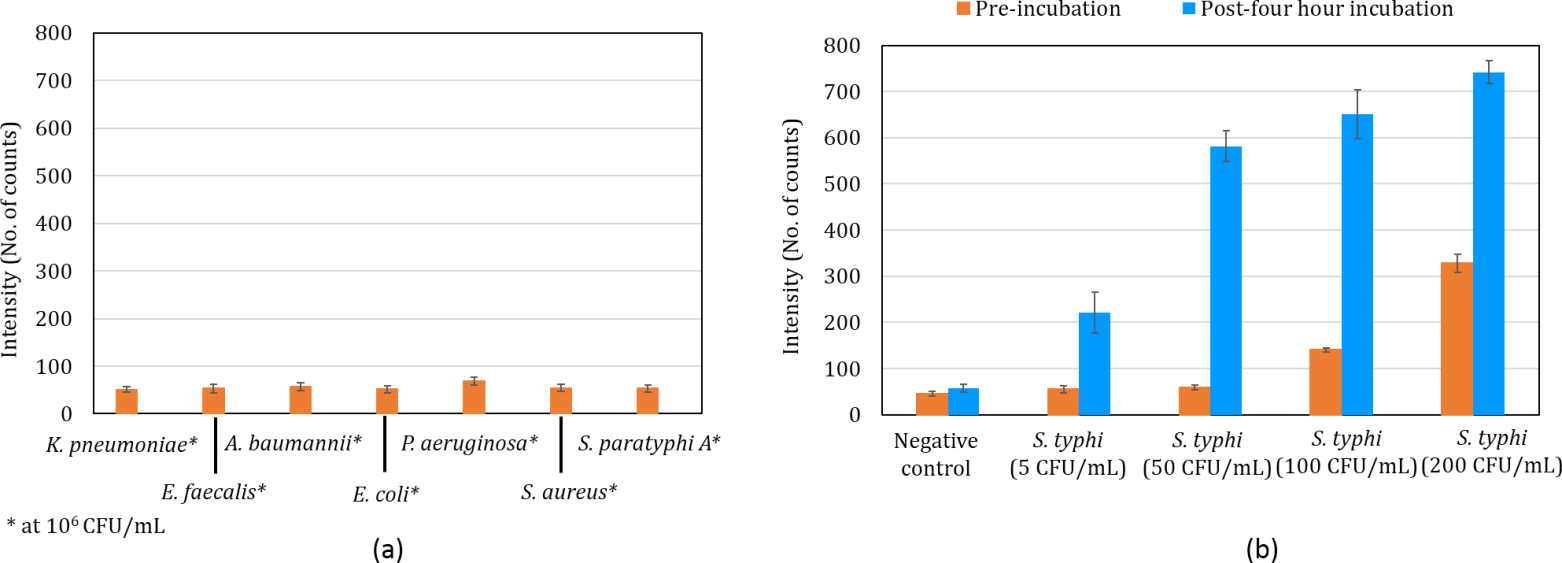
(a) Cross-reactivity of primers against other bacterial species spiked in sterile blood culture media (final concentration of 10^6^ CFU/mL) (b) positive (*S*. *typhi*) and negative control at pre-and post-four-hour incubation.

The cross-reactivity of the primers for the STY2879 gene was examined against *K*. *pneumonia*, *E*. *faecalis*, *A*. *baumanni*, *E*. *coli*, *P*. *aeruginosa*, *S*. *aureus* and *S*. *paratyphi A*. Five microlitres of the DNA template was used for LAMP reaction. The intensity was measured using optical detection system at 520 nm wavelength and 15 minutes. Fig 4(A) represents the cross-reactivity reactions against the other bacterial species as mentioned above. It can be observed that no cross-reactivity was observed even at high concentration of 10^6^ CFU/mL of the pathogenic bacterial species. Fig 4(B) depicts both pre-and post-four-hour incubation of *S*. *typhi* at various concentrations. Though 5 and 50 CFU/mL of *S*. *typhi* shows equivalent signal to negative control and 10^6^ CFU/mL of other bacterial species at pre-incubation, there is a significant increase in the signal for *S*. *typhi* at post-four-hour incubation. In other words, the *S*. *typhi* signals are higher than thrice the signal of negative control post-four-hour incubation.

### Evaluation of clinical samples by *Miod* and conventional method

Assessment of 28 clinical samples by *Miod* and cross-validation with conventional method were performed. In the conventional method, the blood culture bottles were incubated for 24 hours for the given clinical samples at 37°C followed by sub-culture on MacConkey® agar. *S*. *typhi* was confirmed by standard bacteriology methods as shown in S1 Table. The minimum time taken for the conventional method was 72 hours.

**Fig 5:**
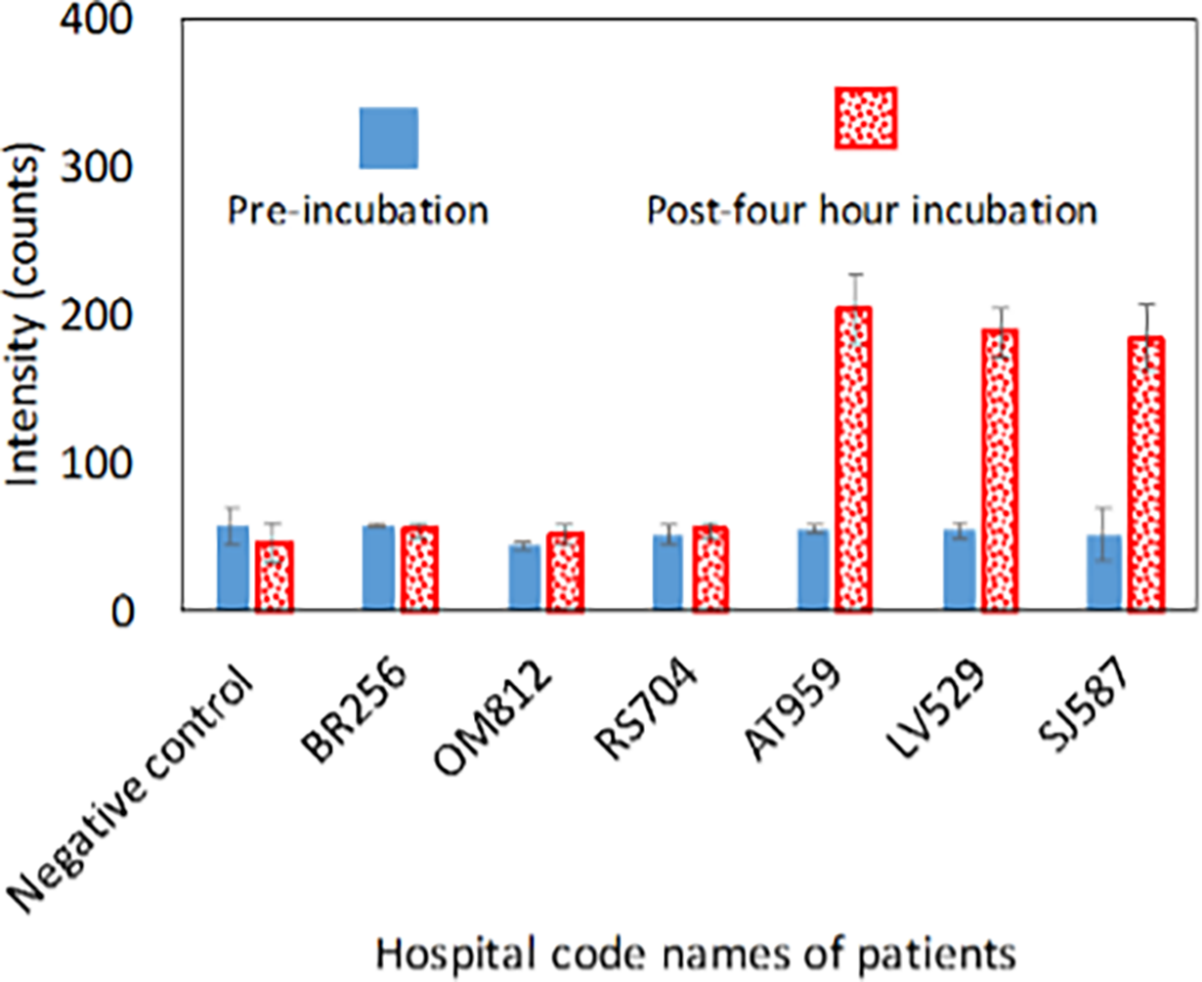
Detection of *S*. *typhi* by *Miod* from human blood samples in the medical institute. The corresponding data are shown only for representative six clinical samples.

Six representative samples including three number of positive and negative samples were identified by *Miod* is shown in Fig 5. The same samples were tested by the conventional method and the detailed observations along with results are given in Table 1. It can be seen that there is a 100% concurrence between the two methods. For the remaining 22 samples, the results of the tests conducted by both *Miod* and the conventional method are given in the S3 Table of the supplementary section. Compared with the conventional method, *Miod*’s clinical sensitivity, clinical specificity, PPV and NPV was 100% (Table 2). Hence, it can be concluded that *Miod* is quite efficient at prediction given its reduced time for diagnosis and at lower LOD. *Miod* confirms the presence of a viable pathogen in the patient within 6 hours as compared to the conventional method of 72 hours that many nuclei acid based devices fail to confirm.

**Table 1:** Results of culture and biochemical tests of the representative six clinical samples.

Clinical sample/ Negative control	Negative control	BR256	OM812	RS704	AT959	LV529	SJ587
Bacterial Culture	No colonies	No colonies	No colonies	No colonies	Formation of pale transparent colonies	Formation of pale transparent colonies	Formation of pale transparent colonies
Motility test	No change	No change	No change	No change	Formation of Red turbidity	Formation of Red turbidity	Formation of Red turbidity
Triple sugar iron test	No change	No change	No change	No change	Red slant and yellow butt	Red slant and yellow butt	Red slant and yellow butt
Citrate test	No change	No change	No change	No change	No change in color	No change in color	No change in color
Urease test	No change	No change	No change	No change	No change in color	No change in color	No change in color
Slide agglutination test	No clumps	No clumps	No clumps	No clumps	Formation of visible clumps	Formation of visible clumps	Formation of visible clumps
Confirmation of *S*. *typhi*	No	No	No	No	Yes	Yes	Yes

**Table 2:** A comparative analysis of conventional method and *Miod* detection of *S*. *typhi*.

Miod	Conventional method Positive	Conventional method Negative	Total	Sensitivity (%)	Specificity (%)	PPV (%)	NPV (%)
Positive	3	0	3	100	100	100	100
Negative	0	25	25				
Total	3	25	28				

Recently, a few techniques have been published on using either optical or colorimetric detection of *S*. *typhi* from spiked blood culture media and human blood samples, after amplification of nucleic acids by LAMP methods. A comparison with of *Miod* with these recently published methods in the literature is given in Table 3. It can be noticed that *Miod* has the lowest LOD of 5 CFU/mL in clinical samples and has comparable processing time. The synergetic effects of magnetic enrichment combined with four-hour incubation and LAMP based amplification protocol helps us in achieving the lowest LOD possible for the given sample. The enrichment using MNP-Ab removes many inhibiting materials and thereby reduces the incidence of false positive and false negative test results. In Table 3, many of the referenced techniques have neither commented on the detection from clinical samples nor on the detection of cell viability. Therefore, it can be observed that *Miod* has the potential for clinical use due to its low LOD, ability to detect viable cells and in a quicker turnaround time. The current device is a proof-of-concept for establishing the ‘*Miod*” protocol. *Miod* has the potential to further be developed into a hand-held, portable and an economical device, however not in the current form. The miniaturization of the device to suit resource-poor settings is the future scope of work.

**Table 3:** Comparison of *Miod* with newly developed LAMP based amplification techniques.

Proposed by	Sample	Volume of sample required	Incubation and processing time	Time for sample preparation, amplification and detection	Confirmation technique of *S*. *typhi*	Sensitivity	Tested on clinical blood samples	Confirms viability of cells
Abdullah et al. [28]	Human blood in blood culture media	100 μL	1 hour	1 hour	Gel electrophoresis	10^4^ CFU/mL	Yes	No
*Miod*	Human blood in blood culture media	2 mL	4 hours	1 hour 40 minutes	Optical detection	5 CFU/mL	Yes	Yes
Ravan and Yazdanparast [26]	Spiked samples in blood	1 mL	2–24 hours	2 hours 40 minutes	ELISA	10 CFU/mL	No	No
Bozorgmehr et al. [27]	Bacterial strains in buffer	200 μL	1 hour	30 minutes	SPR	20 CFU/mL	No	No
Fan et al. [35]	simulated blood and stool samples	2 mL, 500 μL	10 hour	65 minutes	Real-time turbidity	20 CFU/mL (blood), 200 CFU/g (stool)	No	No

## Conclusions

In this work, a rapid and highly sensitive detection of *S*. *typhi* was demonstrated. *Miod* employs a magnetic nanoparticle-based pathogen enrichment protocol, followed by loop-mediated nucleic acid amplification and simultaneous detection by an *in-situ* optical system. The system detected the presence of *S*. *typhi* pre-and post-four-hour incubation in culture media that confirmed cell viability. LOD of the proposed *Miod* was 5 CFU/mL in spiked samples. In the evaluation of human clinical blood samples, *Miod* detected all 28 clinical samples including both positive and negative samples with 100% sensitivity, specificity, PPV and NPV against the conventional methods. The *Miod* protocol including detection was completed in less than 6 hours while the conventional method involving cell culture followed by biochemical confirmatory tests demanded 72 hours or more (S1 Table). Hence, *Miod* has the potential for clinical use due to its low LOD, ability to detect viable cells and in a quicker turnaround time, a combination of significant factors that are not provided by other nucleic acid-based methods. The proposed system be used to detect other pathogens with modification of the primers and minimal modification of the lysis protocol. An offshoot application for the proposed system is the food industry where rapid, on-site testing is necessary to rapidly detect potential sources of contamination and infection. *Miod* shall be miniaturized into a hand-held form factor to adapt to requirements of resource-poor settings. As dehydrated polymerase and primers are stable at room temperature [39], *Miod* platform can be made into a field test device. Due to isothermal amplification, power requirements are minimal and shall be met with portable batteries.

## Supporting information

S1 Fig Typical results observed in biochemical tests: (a) motility test, (b) TSI test, (c) citrate test, (d) urease test. Shown only for comparison between clinical samples (indicated by ‘+’) and control samples (indicated by ‘-‘).
(TIF)

S2 Fig Calibration curve of cell concentration (CFU/mL) versus optical density for *S*. *typhi* bacteria. (TIF)

S1 Table Protocol of conventional method. ^^ indicates tests run in parallel to each other.
(DOCX)

S2 Table Plating of different dilutions of *S*. *typhi* cells spiked in blood culture bottles. 20 μL volume of inoculum was used for the plating.
(DOCX)

S3 Table Results from conventional and proposed *Miod* for 28 clinical samples. (DOCX)

S1 File Supplementary 21st March.docx. (DOCX)
