# Correction: Highly-sensitive detection of *Salmonella typhi* in clinical blood samples by magnetic nanoparticle-based enrichment and *in-situ* measurement of isothermal amplification of nucleic acids

**DOI:** 10.1371/journal.pone.0203982

**Published:** 2018-09-11

**Authors:** Avinash Kaur, Arti Kapil, Ravikrishnan Elangovan, Sandeep Jha, Dinesh Kalyanasundaram

The following information is missing from the Funding section: This study was also funded by the Biotechnology Industry Research Assistance Council (BIRAC), Department of Biotechnology (BIRAC/BT/AIR0275/PACE-12/17).
