# Supplementary figures and images for: Highly-sensitive detection of Salmonella typhi in clinical blood samples by magnetic nanoparticle-based enrichment and in-situ measurement of isothermal amplification of nucleic acids
Source: PLoS One. 2018 Mar 28;13(3):e0194817. doi: 10.1371/journal.pone.0194817 (PMC5874042; doi:10.1371/journal.pone.0194817)

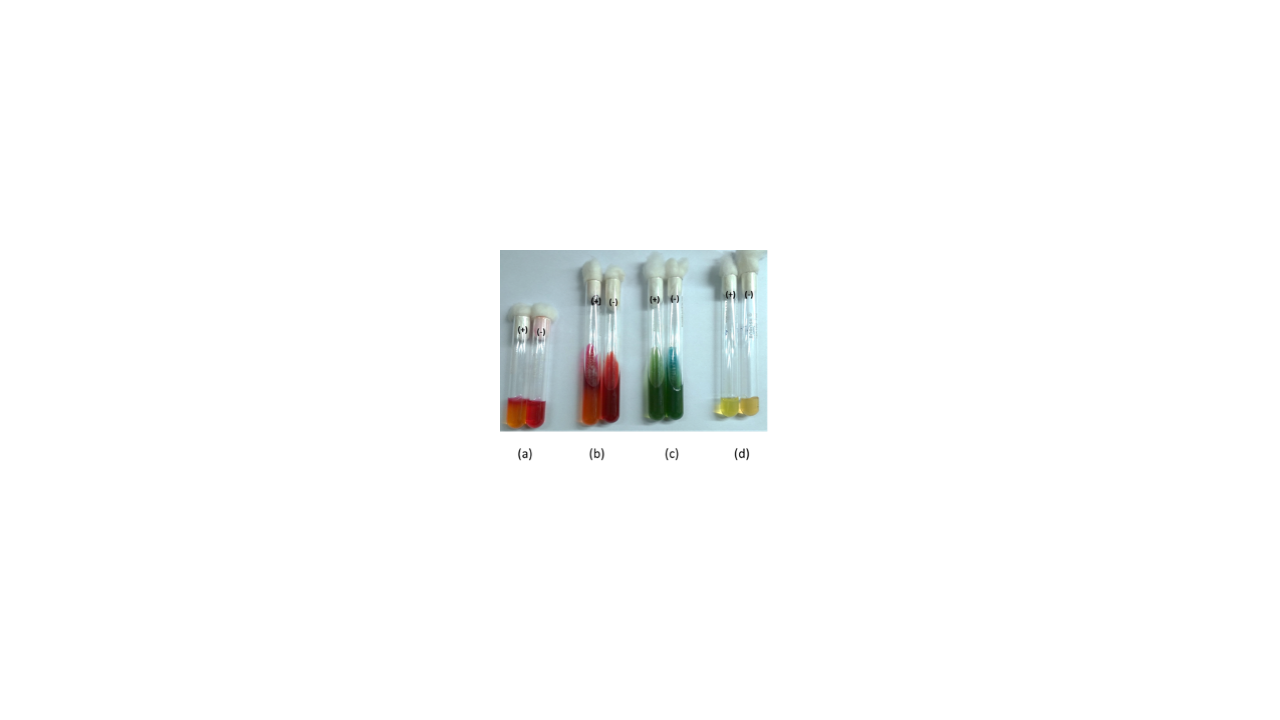

Supplement: S1 Fig — Typical results observed in biochemical tests: (a) motility test, (b) TSI test, (c) citrate test, (d) urease test. Shown only for comparison between clinical samples (indicated by ‘+’) and control samples (indicated by ‘-‘). (TIF) [file pone.0194817.s001.tif]

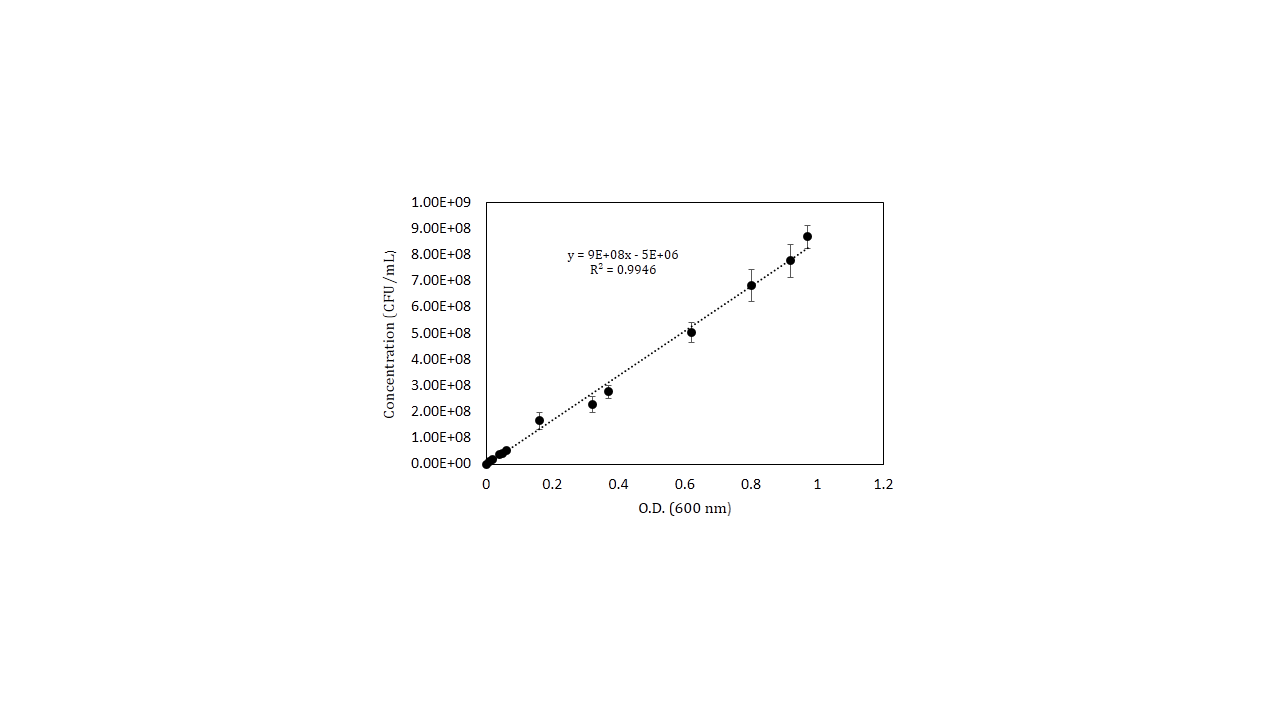

Supplement: S2 Fig — (TIF) [file pone.0194817.s002.tif]
