# Supplementary material for: Highly-sensitive detection of Salmonella typhi in clinical blood samples by magnetic nanoparticle-based enrichment and in-situ measurement of isothermal amplification of nucleic acids
Source: PLoS One. 2018 Mar 28;13(3):e0194817. doi: 10.1371/journal.pone.0194817 (PMC5874042; doi:10.1371/journal.pone.0194817)
Supplement: S1 Table — ^^ indicates tests run in parallel to each other. (DOCX) [file pone.0194817.s003.docx]

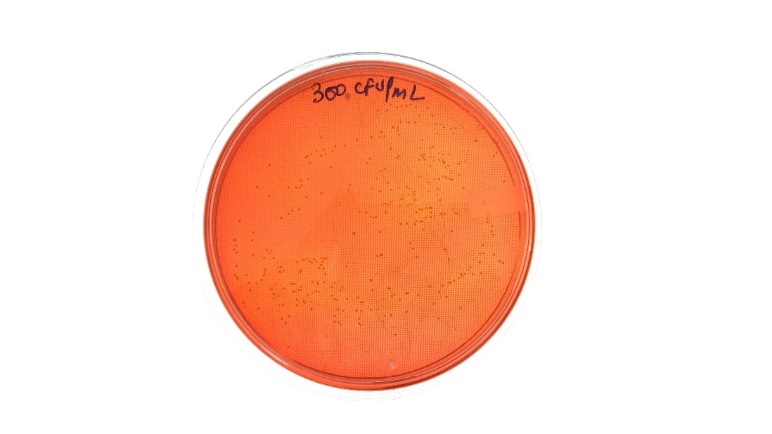
S1 Table. Protocol of conventional method. ^^^^ indicates tests run in parallel to each other.

| Steps | Methods | Initial sample required | Volume of sample required | Time taken | Presence of *S*. *typhi* | References |
| --- | --- | --- | --- | --- | --- | --- |
| 1 | Culture of bacteria in blood culture bottle | Blood sample | 5 mL | 24 hours | Turbid solution |  |
| 2 | Culture of bacteria in Agar plate | Blood culture bottle sample | 3 to 4 µL | 24 hours | Formation of pale transparent colonies | ^32^ |
| 3a | Motility test | Bacterial colonies | 1 to 2 colonies | 24 hours^^^^ | Formation of Red turbidity | ^31^ |
| 3b | Triple sugar iron (TSI) agar | Bacterial colonies | 1 to 2 colonies | 24 hours^^^^ | Red slant and yellow butt | ^31^ |
| 3c | Citrate test | Bacterial colonies | 1 to 2 colonies | 24 hours^^^^ | No change in colour | ^31^ |
| 3d | Urease test | Bacterial colonies | 1 to 2 colonies | 24 hours^^^^ | No change in colour | ^31^ |
| 3e | Slide Agglutination test | Bacterial colonies | 1 to 2 colonies | 2 to 3 minutes^^^^ | Formation of visible clumps | ^31^ |
|  |  |  | **Total time** | **72 hours** |  |  |
