# Supplementary material for: Highly-sensitive detection of Salmonella typhi in clinical blood samples by magnetic nanoparticle-based enrichment and in-situ measurement of isothermal amplification of nucleic acids
Source: PLoS One. 2018 Mar 28;13(3):e0194817. doi: 10.1371/journal.pone.0194817 (PMC5874042; doi:10.1371/journal.pone.0194817)
Supplement: S2 Table — 20 μL volume of inoculum was used for the plating. (DOCX) [file pone.0194817.s004.docx]

S2 Table: Plating of different dilutions of *S*. *typhi* cells spiked in the blood culture media. 20 µL volume of inoculum was used for the plating.

| Pre-incubated samples | No. of colonies formed on Agar plate* from pre-incubated samples (Mean±SD) | No. of colonies formed on Agar plate from post 4 hour incubation* (Mean±SD) |
| --- | --- | --- |
| Negative control | 0 ± 0 | 0 ± 0 |
| 5 CFU/mL | 0 ± 0 | 60×10^3^ ± 21.54×10^3^ |
| 10 CFU/mL | 0 ± 0 | 124×10^3^ ± 27.59×10^3^ |
| 50 CFU/mL | 0 ± 0 | 150×10^3^ ± 27.78×10^3^ |
| 100 CFU/mL | 0 ± 0 | 200×10^3^ ± 47.648×10^3^ |
| 200 CFU/mL | 3 ± 1.73 | 250×10^3^ ± 76.00×10^3^ |
| 300 CFU/mL | 7 ± 2.64 | 280×10^3^ ± 96.46×10^3^ |
| 400 CFU/ml | 14 ± 4.58 | 300×10^3^ ± 147.82×10^3^ |
| 500 CFU/mL | 19 ± 7.54 | 350×10^3^ ± 32.64×10^3^ |

*- Colonies formed post 24 hours of incubation of agar plates
