# Supplementary material for: Highly-sensitive detection of Salmonella typhi in clinical blood samples by magnetic nanoparticle-based enrichment and in-situ measurement of isothermal amplification of nucleic acids
Source: PLoS One. 2018 Mar 28;13(3):e0194817. doi: 10.1371/journal.pone.0194817 (PMC5874042; doi:10.1371/journal.pone.0194817)
Supplement: S3 Table — (DOCX) [file pone.0194817.s005.docx]

S3 Table. Results from conventional and proposed *Miod* for 28 clinical samples

| **S.No** | **Clinical sample** | **Bacterial Culture** | **Motility tests** | **Triple sugar iron test (TSI)** | **Citrate Test** | **Urease test** | **Slide agglutination test** | **Confirmation of *S. typhi* by conventional method** | **Confirmation of *S. typhi* by *Miod* detection** | **Concurrence of results between *Miod* and conventional** |
| --- | --- | --- | --- | --- | --- | --- | --- | --- | --- | --- |
| 1 | Negative control | Negative | Negative | Negative | Negative | Negative | Negative | Negative | Negative | Yes |
| 2 | BR256 | Negative | Negative | Negative | Negative | Negative | Negative | Negative | Negative | Yes |
| 3 | OM812 | Negative | Negative | Negative | Negative | Negative | Negative | Negative | Negative | Yes |
| 4 | RS704 | Negative | Negative | Negative | Negative | Negative | Negative | Negative | Negative | Yes |
| 5 | AT959 | Positive | Positive | Positive | Negative | Negative | Positive | Positive | Positive | Yes |
| 6 | LV529 | Positive | Positive | Positive | Negative | Negative | Positive | Positive | Positive | Yes |
| 7 | SJ587 | Positive | Positive | Positive | Negative | Negative | Positive | Positive | Positive | Yes |
| 8 | KN294 | Negative | Negative | Negative | Negative | Negative | Negative | Negative | Negative | Yes |
| 9 | JD187 | Negative | Negative | Negative | Negative | Negative | Negative | Negative | Negative | Yes |
| 10 | SK806 | Negative | Negative | Negative | Negative | Negative | Negative | Negative | Negative | Yes |
| 11 | DP865 | Negative | Negative | Negative | Negative | Negative | Negative | Negative | Negative | Yes |
| 12 | MJ737 | Negative | Negative | Negative | Negative | Negative | Negative | Negative | Negative | Yes |
| 13 | SD338 | Negative | Negative | Negative | Negative | Negative | Negative | Negative | Negative | Yes |
| 14 | KO450 | Negative | Negative | Negative | Negative | Negative | Negative | Negative | Negative | Yes |
| 15 | VY952 | Negative | Negative | Negative | Negative | Negative | Negative | Negative | Negative | Yes |
| 16 | SD681 | Negative | Negative | Negative | Negative | Negative | Negative | Negative | Negative | Yes |
| 17 | SZ795 | Negative | Negative | Negative | Negative | Negative | Negative | Negative | Negative | Yes |
| 18 | AP776 | Negative | Negative | Negative | Negative | Negative | Negative | Negative | Negative | Yes |
| 19 | RK504 | Negative | Negative | Negative | Negative | Negative | Negative | Negative | Negative | Yes |
| 20 | UR400 | Negative | Negative | Negative | Negative | Negative | Negative | Negative | Negative | Yes |
| 21 | SS752 | Negative | Negative | Negative | Negative | Negative | Negative | Negative | Negative | Yes |
| 22 | LC402 | Negative | Negative | Negative | Negative | Negative | Negative | Negative | Negative | Yes |
| 23 | PK233 | Negative | Negative | Negative | Negative | Negative | Negative | Negative | Negative | Yes |
| 24 | SG900 | Negative | Negative | Negative | Negative | Negative | Negative | Negative | Negative | Yes |
| 25 | MT253 | Negative | Negative | Negative | Negative | Negative | Negative | Negative | Negative | Yes |
| 26 | RK550 | Negative | Negative | Negative | Negative | Negative | Negative | Negative | Negative | Yes |
| 27 | KL877 | Negative | Negative | Negative | Negative | Negative | Negative | Negative | Negative | Yes |
| 28 | DD750 | Negative | Negative | Negative | Negative | Negative | Negative | Negative | Negative | Yes |
