# Supplementary material for: Highly-sensitive detection of Salmonella typhi in clinical blood samples by magnetic nanoparticle-based enrichment and in-situ measurement of isothermal amplification of nucleic acids
Source: PLoS One. 2018 Mar 28;13(3):e0194817. doi: 10.1371/journal.pone.0194817 (PMC5874042; doi:10.1371/journal.pone.0194817)
Supplement: S1 File — (DOCX) [file pone.0194817.s006.docx]

**Supplementary Section**

Highly-Sensitive Detection of *Salmonella typhi* in Clinical Blood Samples by Magnetic Nanoparticle-Based Enrichment and *in-situ* Measurement of Isothermal Amplification of Nucleic Acids

Avinash Kaur^1^, Arti Kapil^2^, Ravikrishnan Elangovan^3^, Sandeep Jha^1, 4^, Dinesh Kalyanasundaram^1, 4*^

^1^ Centre for Biomedical Engineering, Indian Institute of Technology Delhi, New Delhi, India

^2^ Department of Microbiology, All India Institute of Medical Sciences, New Delhi, India

^3^ Department of Biochemical Engineering and Biotechnology, Indian Institute of Technology Delhi, New Delhi, India

^4^ Department of Biomedical Engineering, All India Institute of Medical Sciences, New Delhi, India

* dineshk@cbme.iitd.ac.in, dineshk.iitdelhi@gmail.com

S1.1 Protocol – Conventional method

The detailed conventional protocol of culture followed by confirmatory tests are listed in the S1Table


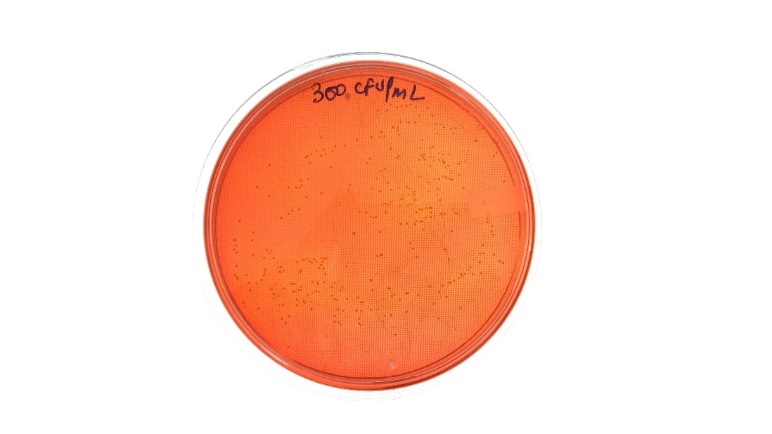
S1 Table. Protocol of conventional method. ^^^^ indicates tests run in parallel to each other.

S1.2 Confirmatory biochemical tests

The motility test for *S. typhi* was confirmed by the formation of red turbidity indicating that the bacteria are motile in nature as compared to negative control. TSI tests showed the formation of red slant and yellow butt indicating as it ferments glucose without the production of gas. The citrate and urease test showed no change in colour as *S. typhi* does not utilize citrate and urease.

The details of the biochemical tests are as follows:

Motility test:

Motility test was used to determine the motility of S. typhi cells. Motility medium consist of triphenyltetrazolium chloride (TTC). The medium is colorless in its oxidized form. As bacteria grows in the presence of TTC, the dye is absorbed into the bacterial cells and therefore the medium is reduced into insoluble red-colored pigment formazan. At first, the medium was stabbed with a sterile needle containing 3 to 4 µL of cells to just above (~1 cm) the bottom of the tube containing motility media. The media was then incubated at 37°C for 18 to 24 hours. A positive motility test was indicated by a red turbid area extending away from the line of inoculation. A negative test is indicated by red growth along the inoculation line.

Triple sugar iron (TSI) agar test:

Triple sugar iron agar test was used to determine whether S. typhi cells utilize glucose, lactose or sucrose and produce hydrogen sulfide (H2S) gas. Phenol red and ferrous sulphate were used as an indicator for acidification of medium and H2S production respectively. Sterile straight wire containing cells was stabbed through the center of the TSI medium to the bottom of the tube and then streaked on the surface of the slant. This was incubated at 37°C for 18 to 24 hours.

Citrate test:

Citrate utilization test was used to determine the ability of S. typhi cells to utilize sodium citrate as a carbon source and inorganic NH4H2PO4 as a source of nitrogen. The bacterial cells that grow in the medium turn the medium alkaline indicated by the change of color of bromothymol from green to blue. The S. typhi cells were inoculated on Simmons citrate agar by streaking on its surface using sterile straight wire and incubated at 37°C for 18 to 24 hours.

Urease test:

Urease test was used to determine whether S. typhi cells produce urease enzyme, an indication of viable cells. The bacterial cells produce urease enzyme, splits urea in the presence of water to release ammonia and carbon dioxide. The ammonia combines with carbon dioxide and water to form ammonium carbonate which turns the medium alkaline, changing the indicator phenol red from its original orange-yellow color to bright pink. The S. typhi cells were inoculated on urease agar medium using sterile straight wire stabbed through the bottom of the tube. The tube was incubated at 37°C for 18 to 24 hours.

Agglutination test:

A slide agglutination test was used to detect S. typhi O9, poly O and H antigens in blood. 10 to 20 µL of saline was poured on to the slide. The colony was picked with the sterile needle and mixed with saline. Antisera containing an antibody specific for O9, poly O and H were added to the slide. The formation of visible clumps observed within 2-3 minutes confirms the presence of *S. typhi.*

S1Fig. Typical results observed in biochemical tests: (a) motility test, (b) TSI test, (c) citrate test, (d) urease test. Shown only for comparison between clinical samples (indicated by ‘**+’**) and control samples (indicated by ‘**-**‘).

S1.3 Calibration curve of cell concentration (CFU/mL) versus optical density for *S. typhi* bacteria.

The measure of CFU/mL calculation using optical density is a standard technique in microbiology in which cells are grown to a particular optical density is serially diluted and plated to count CFU. The same technique has been followed, which includes the calibration curve of cell concentration (CFU/mL) versus optical density at 600 nm for *S.typhi* bacteria is shown in S2 Fig below.

S2 Fig. Calibration curve of cell concentration (CFU/mL) versus optical density for *S. typhi* bacteria

S1.4 CFU count of pre- and post-four hour incubation

*S*. *typhi* cells spiked in sterile blood culture media for different dilutions. The MacConkey® agar was used as the differential media to differentiate between lactose and non-lactose fermenting bacteria. Twenty microliters of each dilution containing *S. typhi* was plated on the agar plate. The plates were incubated for 24 hours and colonies were counted manually. Similarly, post-four hour incubation, *S*. *typhi* cells spiked in a sterile blood culture media were plated on the new set of agar plates (after dilution by 1000 times). The plates were incubated for 24 hours and colonies were counted manually. Table S2 indicates the number of colonies counted pre-and post-four hour incubation.

S2 Table: Plating of different dilutions of *S*. *typhi* cells spiked in the blood culture media. 20 µL volume of inoculum was used for the plating.

S2.0 Results of *S*. *typhi* detection by *Miod* and conventional method

Table S3 shows the complete data of 28 patients with suspected enteric fever. The results were compared with conventional culture method and LAMP based optical detection method. Only three patients showed confirmed *S. typhi* infection using conventional method and LAMP based optical detection methodS3 Table. Results from conventional and proposed *Miod* for 28 clinical samples
